# Induction of Synovitis Using Interleukin-1 Beta: Are There Differences in the Response of Middle Carpal Joint Compared to the Tibiotarsal Joint?

**DOI:** 10.3389/fvets.2018.00208

**Published:** 2018-08-31

**Authors:** Aimee C. Colbath, Steven W. Dow, Leone S. Hopkins, Jennifer N. Phillips, C. Wayne McIlwraith, Laurie R. Goodrich

**Affiliations:** ^1^Department of Clinical Sciences, Orthopaedic Research Center, Colorado State University, Fort Collins, CO, United States; ^2^Clinical Sciences, Colorado State University, Fort Collins, CO, United States

**Keywords:** interleukin-1 beta, carpal, tibiotarsal, horses, synovitis

## Abstract

**Background:** The effects of recombinant interleukin-1β (rIL-1β) have been described for the middle carpal joint (MCJ). However, we are unaware of any studies that have described the cytological response of the tibiotarsal joint (TTJ) to rIL-1β or compared the clinical and cytological responses of the MCJ to the TTJ following the administration of intra-articular rIL-1β. Such information is critical for researchers planning to use rIL-1β to create acute synovitis models in horses.

**Objectives:** To compare the clinical and cytological responses of the MCJ to the TTJ following administration of intra-articular rIL-1β.

**Methods:** Twelve horses were used for the study. Eight horses received 75 ng of rIL-1β into the MCJ and four horses received 75 ng of rIL-1β into the TTJ. Clinical and cytological outcome parameters including lameness, joint circumference, joint effusion score, total nucleated cell count, cellular differentials, C-reactive protein, and prostaglandin-E2 concentrations were determined at baseline and multiple post-treatment time points over a 336 h period (2 weeks).

**Results:** Recombinant IL-1β administered into the TTJ resulted in a significantly greater respiratory rate at 24 h and heart rate at 12 h when compared to rIL-1β administered into the MCJ. In addition, the TTJ had a significantly greater increase in joint circumference at 24 post-injection hour (PIH) and subjective effusion grade at 24 PIH and 336 PIH. The MCJ had significantly higher total protein concentration at 6 PIH, and a significantly higher NCC at 24 and 72 PIH when compared to the TTJ. Conversely, the TTJ had significantly higher neutrophilic infiltration than the MCJ at 6 PIH and 168 PIH.

**Conclusions:** This study establishes that the same intra-articular dose of rIL-1 β elicits significantly different clinical and cytological responses in the MCJ compared to the TTJ in the equine model of intra-articular synovitis. In addition, clinical and cytological evidence of synovitis may persist up to or >1 week following intra-articular administration of rIL-1 β.

## Introduction

Interleukin-1β (IL-1β), an inflammatory cytokine, has been used in multiple *in vivo* and *in vitro* inflammatory models of equine synovitis (1–7). IL-1β has been detected in both human and equine naturally-occurring osteoarthritis (OA), and causes the production of other destructive mediators of OA including matrix metalloproteases (MMPs) and prostaglandin-E_2_ (3, 6, 8, 9). Further, treatments directed at reducing IL-1β, such as interleukin-1 receptor antagonist protein, have resulted in improved clinical outcomes and reduced joint destruction (10–12).

Recombinant interleukin-1β (rIL-1β) produces a reliable, reproducible, short-term synovitis in the equine middle carpal joint (MCJ) (3). The recombinant, equine-specific, cytokine is readily available from a commercial vendor and easily reconstituted for intra-articular administration. A study by Ross et al. (3) comparing the inflammatory response elicited by rIL-1β to that of lipopolysaccharide describes the clinical and cytologic effects of 100 ng of rIL-1β administered into the MCJ. However, an additional study by Toth et al. (4) describing the use of rIL-1β in the stifle reports more severe lameness than described for the MCJ. Further, a study conducted by Carmalt et al. (13) revealed that various joints may respond differently to inflammation.

A recent study (7) and the experiences of the authors of this current study with rIL-1β in the tibiotarsal joint (TTJ) led to the question whether the TTJ may have a different clinical and cytological response to the administration of rIL-1β than described for the MCJ. We felt this was an important question because previous studies have assumed the response to an inflammatory agent is equivalent between MCJ and TTJ and have drawn conclusions regarding the immunomodulatory ability of treatments such as mesenchymal stem cells using TTJ and MCJ as equivalent joints to investigate treatments (14). Further, variability in the TTJ and MCJ joint is important when determining the dose of rIL-1β appropriate for research studies, while comparing treatment responses and evaluating treatment strategies and clinical responses. Therefore, the first objective of the current study was to determine the clinical and cytological response of the TTJ to the administration of 75 ng of rIL-1β. We hypothesized that there would be a cytological response that was reflective of the lameness parameters and that the response would be acute (<3 days). The second objective was to compare the cytological and clinical responses of rIL-1β administered into the TTJ vs. the MCJ. We hypothesized that administration of rIL-1β in the TTJ would result in a greater inflammatory response when compared to the MCJ.

## Materials and methods

### Experimental design

**Figure 1:**
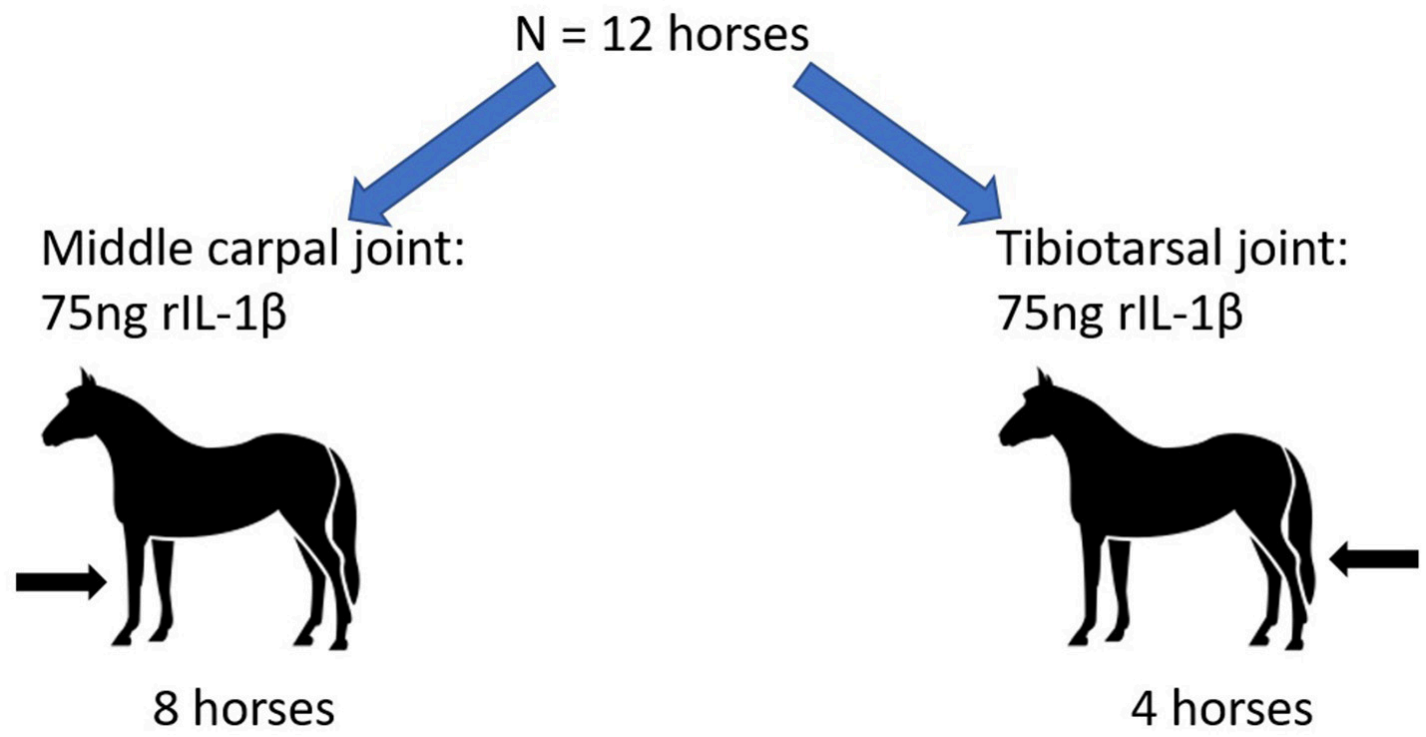
Experimental design. Twelve horses were enrolled in the study. Eight horses received 75 ng rIL-1β into the middle carpal joint. Four horses received 75 ng rIL-1β into the tibiotarsal joint. No other treatments were administered prior to or within 4 weeks following rIL-1β administration.

Twelve horses were utilized for the study. Initial lameness examinations were conducted 2 weeks prior to the start of the study. Eight horses were administered 75 ng of rIL-1β into the MCJ with no other treatment. After a 4 weeks wash out period these same 8 horses entered a subsequent study with administration of rIL-1β and a treatment into the TTJ (data not shown). The investigators became aware that the two joints being investigated, the MCJ and TTJ, may respond differently to the same dose of rIL-1β. Therefore, the investigators designed and executed the current study, comparing the response of MCJ and TTJ to the same dose of rIL-1β alone with no concurrent treatment (Figure 1) four additional horses were administered 75 ng of rIL-1β into a single tibiotarsal joint.

An *apriori* power calculation was performed using Lenthe's power calculator (https://homepage.divms.uiowa.edu/~rlenth/Power/index.html) based on the means and standard deviations for the nucleated cell counts (NCC) and total proteins obtained from the first 8 horses enrolled in the study. The *apriori* power calculation found that four additional horses would produce a power of 0.8, accounting for an alpha error rate of 0.5, if the difference in total protein was 1 gm/dL and the difference in NCC was 34 × 10^3^ cells/uL. When comparing the MCJ and TTJ response to rIL-1β, the initial data suggested a difference in mean NCC of approximately 40 × 10^3^ and a difference in total protein of 1.2 gm/dL. Therefore, 4 additional horses were used to investigate the same dose of rIL-1β administered into the TTJ (without a concurrent treatment) (Figure 1).

All horses were determined to be sound by two ACVS board-certified large animal surgeons on a straight line at the trot prior to enrollment in the study. Horses had no joint effusion present in the MCJ or TTJ and no response to flexion. Horses ranged in age from 2 to 5 years old (mean age: 3.625 years) and were mixed breed. Treatment limbs were randomized using a random number generator (www.random.org), and all investigators and staff were unaware of treatment assignment with the exception of the first author. This work was conducted under the approval of the Institutional Animal Care and Use Committee of Colorado State University (15–5810A). The treatment (75 ng of rIL-1β) were diluted in phosphate buffered saline and administered as 1 ml. All joints were clipped and aseptically prepared before administration of rIL-1β, and treatments were administered using aseptic technique.

### Evaluation of clinical response to treatment

A physical examination including heart rate, respiratory rate and temperature and lameness evaluation was performed, and joints were evaluated for joint circumference, and joint effusion at 0, 6, 12, 24, 72, 168 (1 week), and 336 (2 weeks) post-injection hours (PIH). Subjective lameness examination was conducted by trotting animals, in-hand, and graded using the AAEP lameness scale (https://aaep.org/horsehealth/lameness-exams-evaluating-lame-horse). Subjective lameness was reported as the mean change in lameness for each time point. The change in lameness was calculated for each horse at each time point by subtracting any baseline lameness observed at 0 PIH.

At each time point joint circumference (cm) was measured three times, consecutively, at the same location (at the point of greatest circumference). This location was determined in the normal joint prior to the initiation of the study and marked by clipping hair at the location of measurement. The three values were averaged for each time point. Joint effusion was given a subjective clinical grade with grade 0 indicating no effusion, grade 1 indicating slight effusion, grade 2 indicating mild effusion, grade 3 indicating moderate effusion, and grade 4 indicating severe effusion.

### Synovial fluid analysis

Synovial fluid was harvested prior to treatment (0 PIH) and 6, 12, 24, 72, 168, and 336 PIH. Arthrocentesis was performed aseptically following clinical assessment. Horses were sedated using detomidine hydrochloride (0.01 mg/kg IV) and butorphanol tartrate (0.01 mg/kg IV). Synovial fluid was immediately placed in plain glass tubes and processed within 1 h of collection. A portion of the aspirate was used for direct smear and cytospin analysis prior to hyaluronidase digestion and analysis for total nucleated cell count using an automated cell counter. Total protein content was determined using a refractometer. Differential neutrophil, monocyte, lymphocyte and eosinophil counts were evaluated using direct smear and cytospin analysis. The remainder of the synovial fluid was centrifuged for 10 min at 1,000 × g and the supernatants were stored at −80°C in Eppendorf tubes until ELISA analysis could be performed. Multiple aliquots were frozen to prevent freeze-thaw cycles.

### Enzyme-linked immunosorbent assays

Synovial Prostaglandin E2 (PGE_2_) was evaluated as previously described (3). Briefly, a solid-state extraction was performed using C2 ethyl mini-columns prior to quantification using a commercially available equine specific PGE_2_ Enzyme-linked immunosorbent assay (ELISA) kit^1^^,^^2^. Synovial C-reactive protein was evaluated using a commercially available ELISA kit^3^.

### Statistical analysis

Clinical (subjective and objective lameness, joint circumference, joint effusion) and synovial fluid data (nucleated cell count, total protein, differential cell counts) were compared using a two-way mixed ANOVA for repeated measures with time defined as the within subjects factor, and the joint (TTJ vs. MCJ) defined as a between-subjects effect. Significance was set at *P* < 0.05. Simple effects between treatments were analyzed using a Tukey's multiple comparisons test. Normality was assessed by evaluating diagnostic plots of the residuals for each variable. Log transformation was performed for nucleated cell count data. Statistical analysis was conducted using the R “lsmeans” statistical package (version 3.3.3).

## Results

### Clinical responses

**Figure 2:**
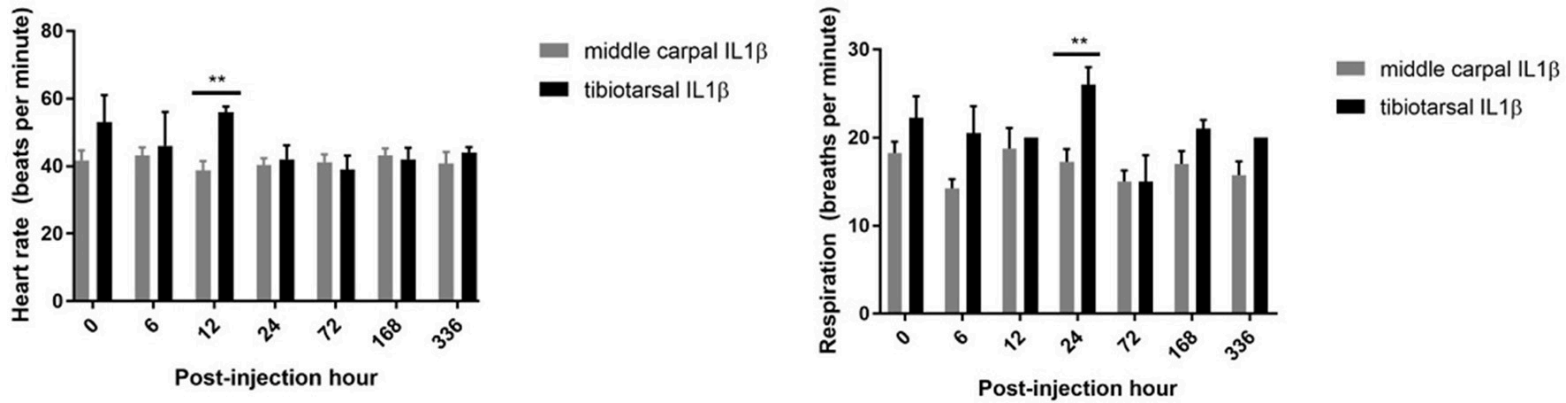
Heart rate and respiratory rate of horses prior to and following rIL-1β administration into the middle carpal joint and tibiotarsal joint. An increase in mean heart rate and respiratory rate is seen in horses receiving rIL-1β into the TTJ at 12 and 24 h, respectively, when compared to the MCJ. Error bars represent that standard error of the mean (SEM) and significance is indicated by **(*P* < 0.01).

Physical examination parameters (heart rate, respiratory rate and temperature) were measured at each time point. Although temperature was not different between groups, rIL-1β administered into the TTJ resulted in a greater respiratory rate at 24 h (*P* = 0.0013) (mean, MCJ: 17 bpm vs. TTJ: 26 bpm) and a greater heart rate at 12 h (*P* = 0.0018) (mean, MCJ: 38 bpm vs. TTJ: 56 bpm) when compared to horses receiving rIL-1β in the MCJ (Figure 2).

**Figure 3:**
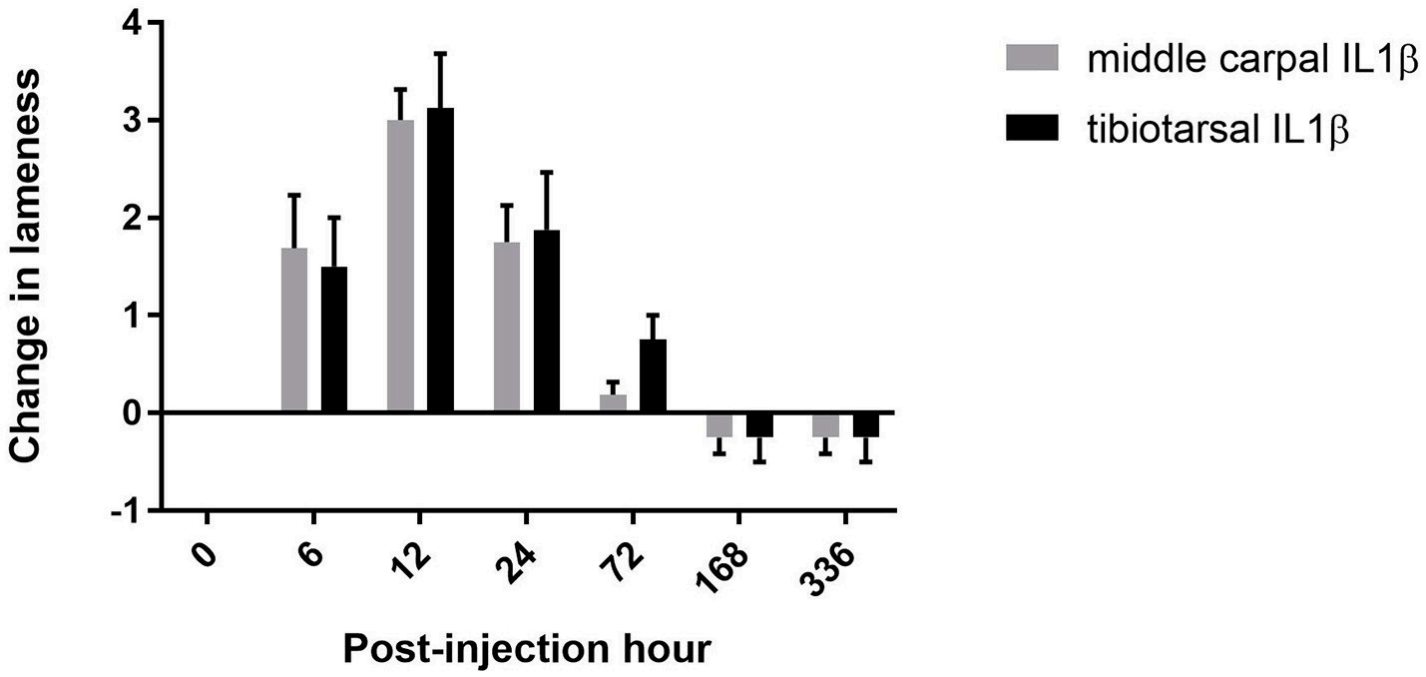
Change in subjective lameness score. There was no difference detected in the mean change of AAEP lameness scores when horses were administered rIL-1β in the MCJ or TTJ. Error bars represent the SEM.

Although horses were evaluated for lameness (in-hand at the trot) 2 weeks prior to starting the study and determined to be sound by two ACVS board certified large animal surgeons using the AAEP grading scale, one horse in each group was found to have a grade 1 lameness at baseline. Therefore, each horse's lameness was calculated at each timepoint as a change in AAEP lameness grade from baseline. Interestingly, both horses with a grade 1/5 lameness at baseline were found to have no lameness 2 weeks following rIL-1β administration. Therefore, change in lameness for these horses was reported as a value of “−1” at 168 PIH. In all horses administered rIL-1β into the MCJ, subjective lameness scores increased by 6 PIH (*P* = 0.0013) (mean change, MCJ: 3). In contrast, horses administered rIL-1β into the TTJ showed a significant increase in subjective lameness by 12 PIH compared to baseline measurements (*P* < 0.0001) (mean change, TTJ: 3.12). Lameness continued above baseline, for both groups, until 72 h post-injection. There was no difference between the change in lameness when rIL-1β was administered in the MCJ vs. the TTJ at any time point (Figure 3).

The mean joint circumference at baseline for the MCJ and TTJ were 26.81 ± 0.912 cm and 31.12 ± 1.46 cm, respectively. The change in joint circumference was measured over time for both groups. Both treatment groups demonstrated an increase in joint circumference by 72 PIH (mean change, MCJ: 2.07 cm (*P* = 0.0233) vs. TTJ: 2.96 cm, (*P* = 0.0036)) with the TTJ showing increased joint circumference at 24 PIH (*P* = 0.002) (mean change, TTJ: 3.75 cm). Change in joint circumference was greater for the horses administered rIL-1β into the TTJ at 24 h when compared to horses administered rIL-1β into the MCJ (Table 1; mean change, MCJ: 1.21cm vs. TTJ: 3.75 cm) (*P* = 0.0015). For both treatment groups, an increase in subjective effusion grade was noted at 6 PIH (*P* < 0.05; mean change, MCJ: 1.75 (*P* < 0.0001) vs. TTJ: 1.50 (*P* = 0.0017)). Horses receiving rIL-1β into the TTJ had a greater change in subjective effusion grade vs. the MCJ at 24 PIH (Table 1) (mean change, MCJ: 2.07 vs. TTJ: 3.25; *P* = 0.0096) and 336 PIH (mean change, MCJ: 2.50 vs. TTJ: 1.25; *P* = 0.0274). A temporal summary of all the clinical results may be found in Supplemental Information 1.

**Table 1:** Joint circumference and effusion scores following rIL-1β administration.

	**Middle carpal joint mean (±*SD*)**	**Tibiotarsal joint mean (±*SD*)**	***P*-value**
**CHANGE IN EFFUSION SCORE**			
0 PIH	0 (± 0)	0 (0 ± 0)	1.0
6 PIH	1.75 (± 0.46)	1.5 (± 0.58)	0.58
12 PIH	2.38 (± 0.52)	3.0 (± 0.82)	0.16
24 PIH	2.06 (± 1.08)	3.25 (± 0.50)	0.01*
72 PIH	1.25 (± 0.89)	2.0 (± 0)	0.09
168 PIH	1.0 (± 1.07)	1.25 (± 0.50)	0.57
336 PIH	0.25 (± 0.89)	1.25 (± 0.50)	0.03*
**CHANGE IN CIRCUMFERENCE (CM)**			
0 PIH	0 (0–0)	0 (0–0)	1.0
6 PIH	0.65 (± 0.54)	0.41 (± 1.7)	0.74
12 PIH	0.94 (± 0.57)	1.52 (± 1.93)	0.44
24 PIH	1.21 (± 1.17)	3.75 (± 2.03)	0.002**
72 PIH	2.07 (± 2.53)	2.96 (± 0.98)	0.24
168 PIH	0.91 (± 0.68)	1.97 (± 0.78)	0.16
336 PIH	0.45 (± 0.56)	1.55 (± 0.95)	0.15

Significant differences between the MCJ and TTJ are noted by

* (P < 0.05) and

** *(P < 0.01)*.

### Synovial fluid analysis

**Figure 4:**
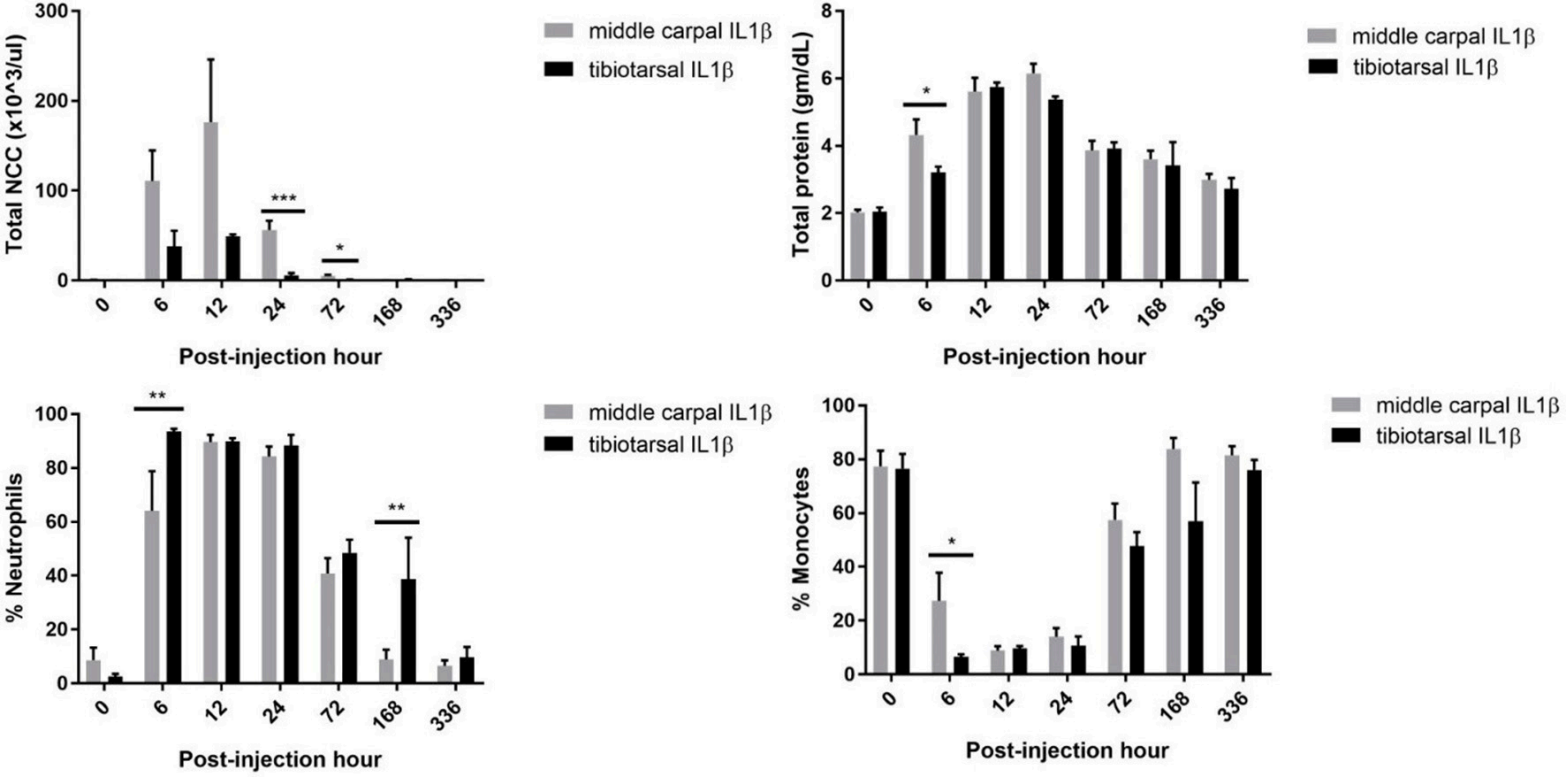
Cytologic analysis following IL1β administration. Total nucleated cell count (NCC) was higher at 24 PIH and 72 PIH when rIL-1β was administered into the MCJ. In contrast, the percent of neutrophils was increased in the TTJ when compared to the MCJ at both 6 PIH and 168 PIH. Error bars represent SEMs of the mean and significance is indicated by *(*P* < 0.05), **(*P* < 0.01), and ***(*P* < 0.001).

Synovial fluid was analyzed for total nucleated cell count (NCC) and total protein, and percent neutrophils, monocytes, lymphocytes, and eosinophils were calculated using a differential cytology determined by cytospin or direct smear. Six of eight horses' receiving rIL-1β in the MCJ had a NCC peak at 6 h and the remaining two horses peaked at 12 h. All horses that received rIL-1β into the TTJ had a NCC peak at 12 PIH. The NCC was higher in the MCJ at 24 PIH (*P* = 0.0005; mean NCC, MCJ: 56.25 × 10^3^/μl vs. TTJ: 5.96 × 10^3^/μl) and 72 PIH (*P* = 0.04; mean NCC, MCJ: 5.03 × 10^3^/μl vs. TTJ: 0.98 × 10^3^/μl) when compared to the TTJ joint (Figure 4). Despite a higher NCC in the MCJ, neutrophilic infiltration occurred faster in the TTJ resulting in a significantly larger percentage of neutrophils in the TTJ vs. MCJ at 6 PIH (*P* = 0.007; % neutrophils, MCJ: 64.13% vs. TTJ: 93.50%). Likewise, the monocytic population remained higher in the MCJ synovial fluid vs. the TTJ synovial fluid at 6 PIH (*P* = 0.0264; % monocytes, MCJ: 27.37% vs. TTJ: 6.50%) (Figure 4). In addition, the TTJ experiences a longer duration of neutrophilic inflammation resulting in a significantly greater percentage of neutrophils at 168 PIH (1 week) vs. the MCJ (*P* = 0.0061; % neutrophils, MCJ: 8.88% vs. TTJ: 38.75%). The total protein increased faster in the MCJ, resulting in a significant increase from baseline at 6 PIH (*P* < 0.0001). Conversely, a significant increase in total protein was not detected in the TTJ until 12 PIH (*P* < 0.0001). The total protein in the MCJ was significantly greater than that of the TTJ at 6 PIH (*P* = 0.0228; mean total protein, MCJ: 4.33 g/dL vs. TTJ: 3.20 g/dL; Figure 4). A temporal summary of the pertinent synovial fluid analysis results may be found in Supplemental Information 1.

### Synovial fluid biomarkers

**Figure 5:**
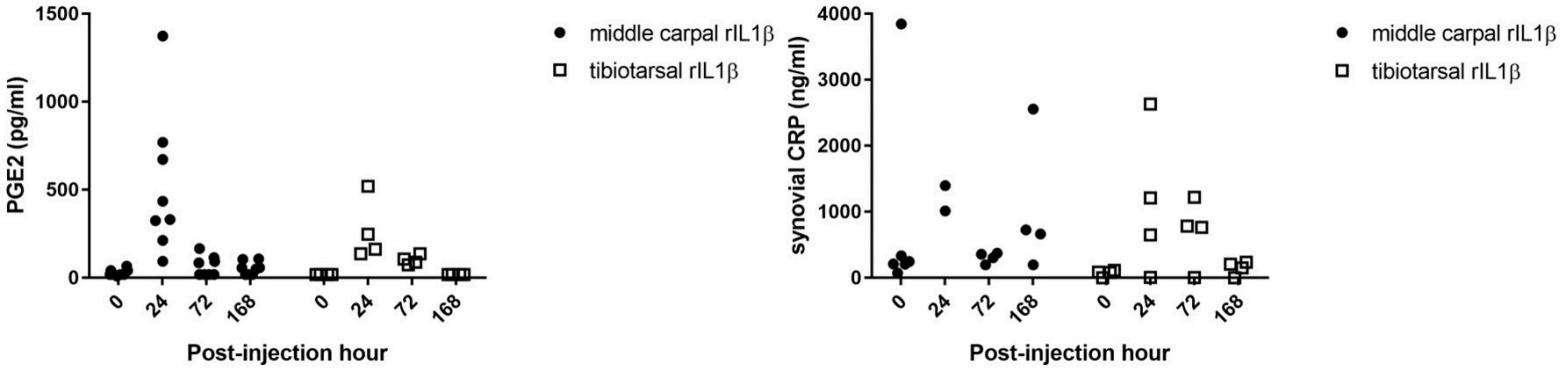
Synovial fluid biomarkers. There was no significant difference in synovial PGE_2_ or synovial CRP levels between MCJ and TTJ at any time point.

No significant differences were detected in synovial fluid levels of PGE_2_ and C-reactive protein between treatment groups (Figure 5).

## Discussion

This study was performed to clarify differences between injecting equivalent doses of rIL-1β in the TTJ compared to the MCJ because subjectively, a previous report as well as clinical observations by the authors of the current study, suggested that these joints may respond differently to the same dose of rIL1-β (7). Further, no other reports reveal the longitudinal, clinical and cytological changes that occur without intervention (such as joint lavage, or biopsies) when rIL-1β is administered into the MCJ or TTJ. The results of this work highlight the differences between the response to rIL-1β in commonly studied joints used for modeling synovitis and provide a reference of respective joint and systemic reactions to rIL-1β. Although synovial biopsies and arthroscopic examination would have provided additional information (3, 7), they also require invasion of the joint capsule and/or joint lavage which could significantly change the cytological parameters measured. Therefore, we excluded these procedures to obtain a 2-weeks assessment of clinical and cytological findings without confounding results with biopsy or surgical lavage which would be used to assess gross and histological changes in response to rIL-1β.

The present study revealed the greatest increase in both TTJ and MCJ circumference (synovial effusion) was at 24 PIH. In contrast, a recent study that utilized standing arthroscopy to perform biopsy samples 10 h following administration of rIL-1β into the tibiotarsal joint reported a decrease in synovial effusion at 24 PIH (following arthroscopic biopsy) when compared to 4 PIH. Without arthroscopic lavage, the current study demonstrated the maximum increase in effusion score for both TTJ and MCJ was at 24 h. Additionally, significant (*P* < 0.05) effusion was detected as late as 336 PIH in the tibiotarsal joint. This finding was unexpected as other studies report a shorter period of post injection effusion (3, 7). Likewise, neutrophilic inflammation was still present in the tibiotarsal joint at 168 PIH. Therefore, previous studies (3, 7) may have underestimated the effect of rIL-1β on the duration of neutrophilic inflammation due to surgical lavage that was performed to assess the joint.

Interleukin-1 beta promotes multiple inflammatory mediators including nitric oxide, PGE_2_, chemokines, adhesion molecules, matrix metalloproteinases, and multiple cytokines leading to synovitis, cartilage destruction and ultimately osteoarthritis. (3, 6, 8, 9) There is significant precedent for the use of rIL-1β to induce inflammation *in vitro* assays (6, 15–17); however, only a few studies have reported the utility of rIL-1β for *in vivo* studies. (2–4, 7, 18) Ross et al. (3) provided the first description of rIL-1β to induce acute synovitis in the MCJ of the horse. Four additional horse studies have followed; one that also utilized the carpus, two which sought to induce acute synovitis in the equine stifle, and a recent study using rIL-1β in the tibiotarsal joint. (2, 4, 7, 18) The first study used 100 ng of rIL-1β in the joint in the carpus, (3) while a pilot study used 100 ng of rIL-1β in the stifle, (2) and later, the same group conducted a study using 200 ng of rIL-1β in the stifle. (4) Most recently, a study initially used 100 ng of rIL-1β in the tibiotarsal joint prior to reducing the dose to 50 ng (7). No studies have compared different joint responses within the same individual to equivalent doses of rIL-1β.

The TTJ, like the MCJ, has distinct advantages for joint studies, including its accessibility, and a large volume of synovial fluid for sampling. A previous study compared the TTJ to the MCJ to analyze joint responses to a therapeutic intervention (stem cells) following injection of an inflammatory agent (14). This study made the assumption that the TTJ and the MCJ would react similarly to lipopolysaccharide. However, our study indicates that it would be inappropriate to assume that the MCJ and the TTJ would respond similarly to an inflammatory agent. This is important when determining both study design and drawing conclusions with regard to intra-articular treatments based on cytological differences. The study also reinforces the importance in determining individual joint responses to an inflammatory agent such as rIL-1β or lipopolysaccharide.

No differences were measured between the change in subjective lameness when rIL-1β was administered in the TTJ and MCJ. A *post-hoc* sample size calculation supports an equivalent lameness between treatment groups, as over 1,200 horses would be needed to find a one-degree difference in subjective lameness using the observed standard deviation with 80% power. But, horses in which the TTJ was injected with rIL-1β, had a significantly higher heart rate and respiratory rate at 12 and 24 PIH than horses receiving rIL-1β in the MCJ which may indicate an increased pain level. Limitations in the range of values (0–5) within the AAEP scale may have decreased our ability to detect more subtle differences. Despite the limitations of lameness scale, our study supports a similar duration (72 h) and a degree of lameness between the TTJ and MCJ when the AAEP lameness grading scale is used.

Recombinant IL-1β is known to cause a substantial synovitis characterized by rapid neutrophilic infiltration (3). The level of neutrophilic inflammation has been described in the MCJ but no other joints (3) Our study is the first to characterize the cytologic response of the TTJ to rIL-1β for 336 PIH and further, to report responses without interceding with joint lavage and/or cartilage and synovial biopsies. We highlight here how the MCJ and TTJ responded differently to the same dose of rIL-1β and provide researchers data concerning the responses of the MCJ and the TTJ to rIL-1β. Finally, this may also suggest how the TTJ and MCJ may respond differently in the clinical setting to acute, non-septic, inflammation or how joint type may affect the progression of osteoarthritis.

Total cellular infiltration as a result of rIL-1β administration was significantly lower in the TTJ compared to the MCJ at 12 h PIH. The sampling times of the current study were slightly different than those performed previously by Ross et al. (3). However, the previous study of the MCJ found a mean NCC at 4 PIH (134.30 × 10^3^) and 8 PIH (170 × 10^3^), Ross et al. (3) similar to those reported here for 6 PIH (110.60 × 10^3^) and 12 PIH (176.15 × 10^3^). As expected from previous studies, the increased NCC is a result of neutrophil infiltration into the joint, where neutrophils compose greater than 70% of the MCJ NCC at 12 h, and greater than 90% of the TTJ NCC at 12 h. In the results, we reported both total NCC and the percentage of each cell type instead of reporting total differential cell counts. This was done as reporting total cell numbers for differential cell types such as neutrophils or monocytes would have disguised an important difference between groups. Namely, the percent neutrophils were higher in the TTJ despite a lower NCC. By 24 h, the NCC was statistically and substantially higher in the MCJ vs. the TTJ (mean NCC, MCJ: 56.25 × 10^3^ μl vs. TTJ: 5.96 × 10^3^/μl) and stayed consistently higher through 72 PIH (mean NCC, MCJ: 5.03 × 10^3^/μl vs. TTJ: 0.98 × 10^3^/μl). Although the TTJ had a lower total NCC compared to the MCJ, a greater percentage of neutrophils composed the inflammatory infiltrate in the TTJ at 6 PIH and 168 PIH. In summary, there was a higher percentage of neutrophils but lower total NCC in the TTJ compared to the MCJ. This may be attributed to an increased synovial fluid produced in the TTJ. The TTJ had a more rapid increase in joint circumference than the MCJ and a greater increase in joint circumference at 24 PIH. Likewise, the subjective joint effusion scores of the TTJ were significantly higher than the MCJ at 24 PIH. Synovial fluid, an ultrafiltrate, likely caused a “dilutional” effect in the TTJ resulting in a decreased total NCC despite a higher percentage of neutrophils.

The MCJ has a synovial continuation with the carpometacarpal joint and the TTJ has a synovial continuation with the proximal intertarsal joint. The TTJ appears to accommodate a larger volume of fluid then the MCJ (Colbath AC, *unpublished data*). Although both the MCJ and TTJ have dorsal and palmar/plantar extensions, the palmar extension of the MCJ is firmly attached to the third carpal bone. Both the dorsal and palmar/plantar pouches of the MCJ and TTJ are lined by synovium. The volume of the MCJ and TTJ have not been compared in the literature. However, in one study, arthrocentesis of the TTJ resulted in 6.25–21 ml of synovial fluid (mean: 10 ml ± 1.2 ml) (19). Our clinical and arthroscopic experience indicates that the tibiotarsal joint has a larger joint volume and greater synovial lining pliability when compared to the MCJ. Interleukin-1β results in the production of many cytokines produced by synoviocytes including interleukin-8 which is a chemokine that initiates neutrophilic activation and recruitment (20, 21) The larger TTJ joint pouch lending to greater synovial surface area, may result in larger amounts of subsequent neutrophilic migration into the joint. In addition to differences in the NCC between the MCJ and TTJ, the MCJ had a faster increase in total protein and a greater total protein at 6 PIH when compared to the TTJ. Again, this could be explained by a greater increase in synovial fluid, an ultrafiltrate, in the TTJ when compared to the MCJ.

The initial volume of the TTJ may be greater than the MCJ for the same dose of rIL-1β; however, the change in lameness is similar. Conversely, physical examination characteristics (heart rate and respiration) suggest potentially greater pain associated with rIL-1β administration in the TTJ. The increase in pain may be explained by increased synovial fluid production, leading to an increase in joint circumference and effusion resulting in stretching of the joint capsule and a greater pain response from joint distention.

Different cohorts of horses were utilized instead of a washout model, as previous equine rIL-1β studies had not established the duration of effect without biopsy or lavage. Synovial biopsies were not taken during the study period. However, two horses that were administered rIL-1β into the TTJ were euthanized for a different study and synovial biopsies were taken at the time of euthanasia, approximately 98 days post-injection. At the time of necropsy, one horse had an increased synovial cellular infiltration, intimal hyperplasia, and subintimal fibrosis compared to the un-injected TTJ. These results would indicate a model employing a “washout period” may be inappropriate unless the washout period is lengthy or joint lavage is performed.

All horses received the same dose of rIL-1β. This was done to provide a comparison between the joint response to the same dose of rIL-1β. Alternatively, the dose could have been titrated to the estimated volume of the joint but this would be difficult and was beyond the scope of this study. All rIL-1β in this study was from the same lot and stored and reconstituted identically. This is important as different lots and methods of storage and reconstitution may lead to varying activity levels (3, 4, 7). A future study could also compare the response of both TTJ and MCJ to a dose escalation of rIL-1β.

Although an *apiori* power calculation was performed and our sample size was adequate to detect statistical differences in both clinical (other than lameness) and cytological parameters including heart rate, respiratory rate, joint effusion and differential cell counts, the small sample size remains a limitation of the study. However, due to the small standard deviations in the observed cytological and clinical parameters, *post-hoc* power calculations revealed the statistical power to exceed 80% for all parameters excluding heart rate and total protein concentration. Further, the *post-hoc* power calculation for total protein exceeded 70%.

In conclusion, we had hypothesized that administration of rIL-1β in the TTJ would result in an acute (< 3 days) cytological and clinical response and that inflammation would be greater in the TTJ when compared to the MCJ. Our hypotheses were partially correct; the inflammation could not be characterized as acute. However, the TTJ does have a longer-lasting inflammatory response characterized by greater neutrophilic inflammation when compared to the MCJ. Although lameness subsided within 3 days, neutrophilic inflammation persisted in the TTJ (and was significantly greater than the MCJ) at 1-week post-injection, and effusion was still detectable in the TTJ at 2 weeks post-injection. These results indicate that a >2 weeks washout period is necessary when administering rIL-1β into the TTJ. Although the TTJ experienced a longer duration of effusion and neutrophilic inflammation, the total NCC were lower in the TTJ at 24 and 72 PIH when compared to the MCJ. This study provides important clinical and cellular parameters for future investigations in which researchers plan to utilize rIL-1β in an equine model of intra-articular inflammation. Previous studies have used the MCJ as a control for treatments administered into the TTJ (14). The current study provides evidence of varying cytological responses between the TTJ and MCJ and suggests that these joints should not be considered similar in the clinical and cytological responses. In addition, this is the first study to describe the clinical effects, cytology, total protein, and inflammatory mediators resulting from the administration of rIL1β into the equine TTJ or MCJ for 336 PIH.

## Author contributions

AC, LG, SD, and CM were involved in planning the study and interpreting the results. AC collected all samples, performed all clinical assessments, drafted the manuscript and designed the figures. AC, LH, and JP processed the experimental data. All authors were involved in manuscript revisions.

### Conflict of interest statement

The authors declare that the research was conducted in the absence of any commercial or financial relationships that could be construed as a potential conflict of interest.

## Footnotes

^1^R&D Systems, Minneapolis, Minnesota.

^2^Enzo, Farmingdale, NY.

^3^Immunology Consultants Laboratory, Portland, OR.

## Abbreviations

MCJ: middle carpal joint
NCC: nucleated cell count
PIH: post-injection hour
rIL-1β: recombinant interleukin-1 beta
TTJ: tibiotarsal joint.
