# Supplementary material for: Induction of Synovitis Using Interleukin-1 Beta: Are There Differences in the Response of Middle Carpal Joint Compared to the Tibiotarsal Joint?
Source: Front Vet Sci. 2018 Aug 31;5:208. doi: 10.3389/fvets.2018.00208 (PMC6127273; doi:10.3389/fvets.2018.00208)
Supplement: Supplementary file 1 [file Data_Sheet_1.DOCX]

**Supplement 1.** This document is provided as a reference to researchers. The document summarizes clinical (change in lameness, change in joint effusion, change in joint circumference), and synovial (total protein, nucleated cell count, percent neutrophils) findings after injection of 75ng of rIL-1β into the middle carpal joint and tibiotarsal joint for up to 336 post-injection hours.


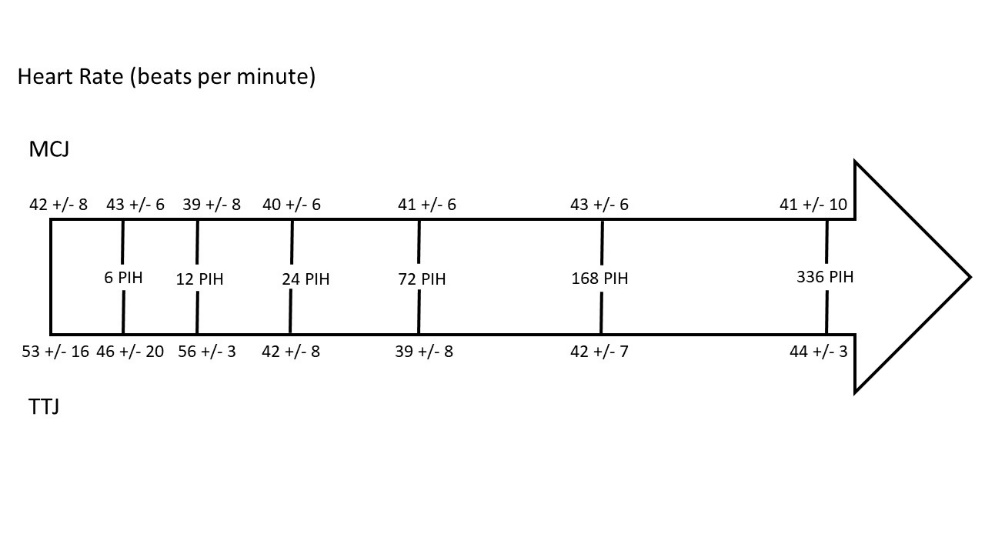


**Figure 1.** Progression of heart rate (beats per minute, mean +/- standard deviation) changes over 336 post-injection hours after administration of 75ng rIL-1β into the middle carpal joint (MCJ) and tibiotarsal joint (TTJ) of the horse.


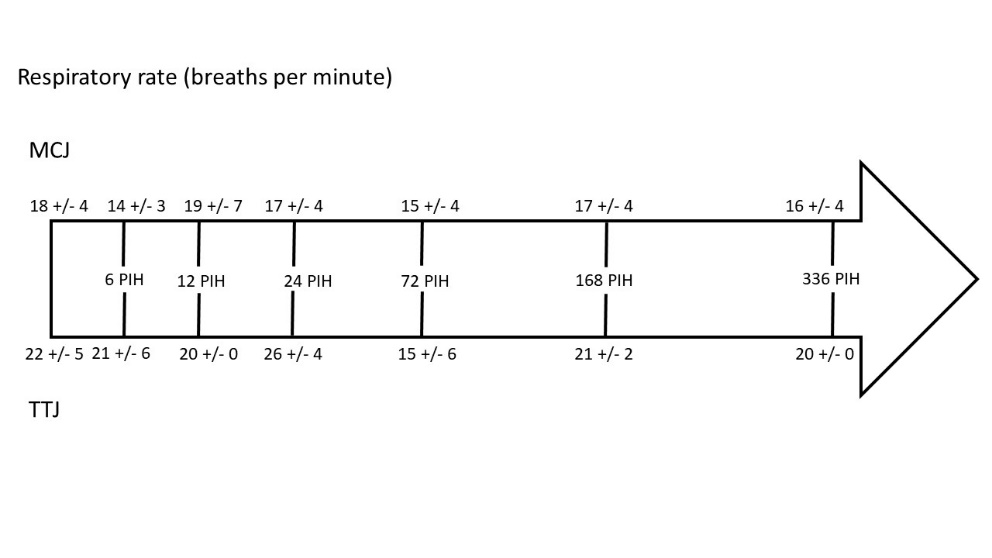


**Figure 2.** Progression of respiratory rate (breaths per minute, mean +/- standard deviation) changes over 336 post-injection hours after administration of 75ng rIL-1β into the middle carpal joint (MCJ) and tibiotarsal joint (TTJ) of the horse.


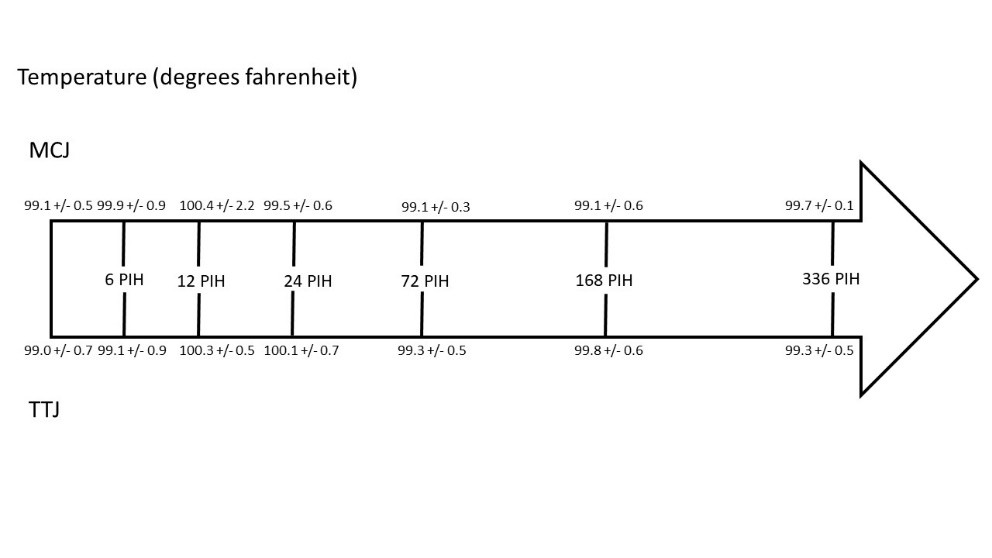


**Figure 3.** Progression of temperature (degrees Fahrenheit, mean +/- standard deviation) changes over 336 post-injection hours after administration of 75ng rIL-1β into the middle carpal joint (MCJ) and tibiotarsal joint (TTJ) of the horse.


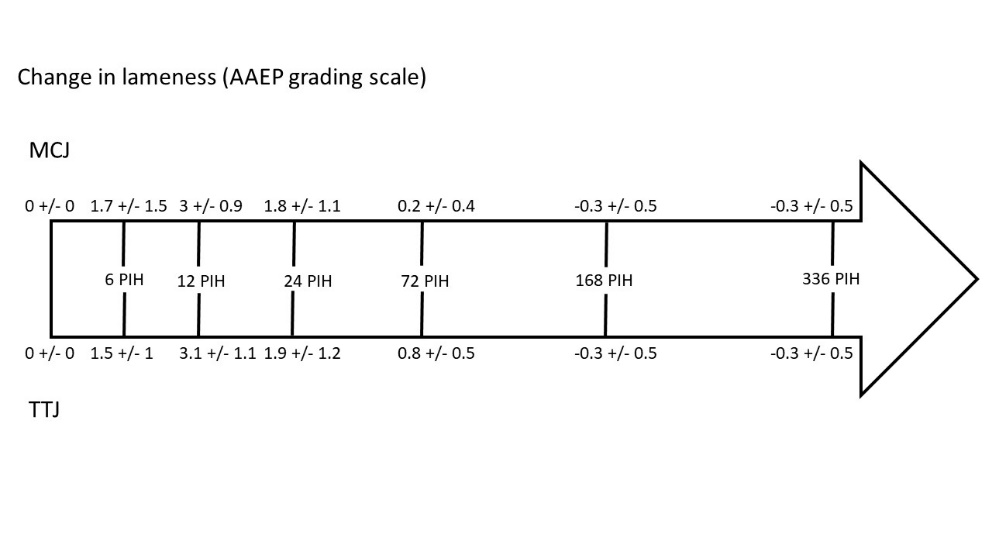


**Figure 4.** Progression of change in lameness grade (AAEP grading scale, mean +/- standard deviation) changes over 336 post-injection hours after administration of 75ng rIL-1β into the middle carpal joint (MCJ) and tibiotarsal joint (TTJ) of the horse.


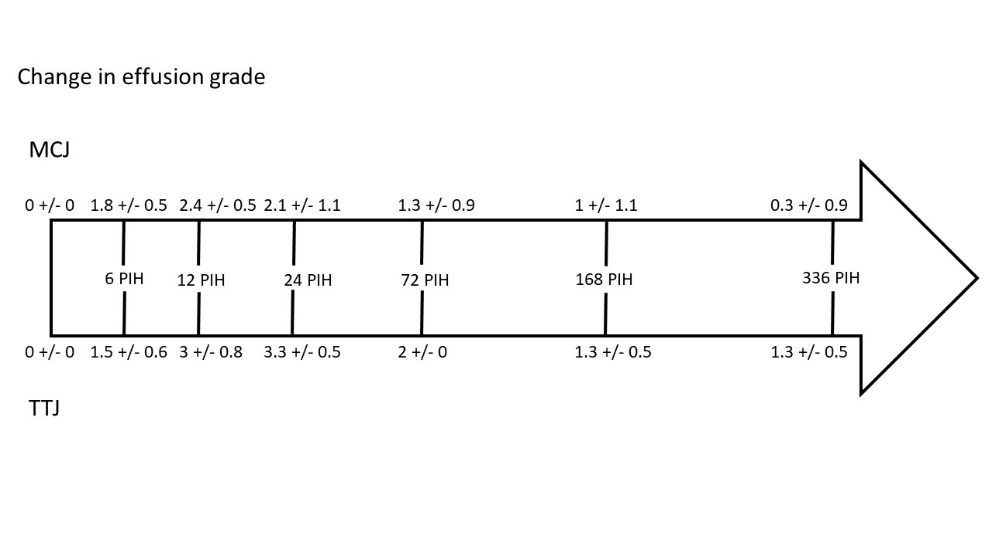


**Figure 5.** Progression of change in effusion grade (subjective effusion grading scale, mean +/- standard deviation) changes over 336 post-injection hours after administration of 75ng rIL-1β into the middle carpal joint (MCJ) and tibiotarsal joint (TTJ) of the horse.


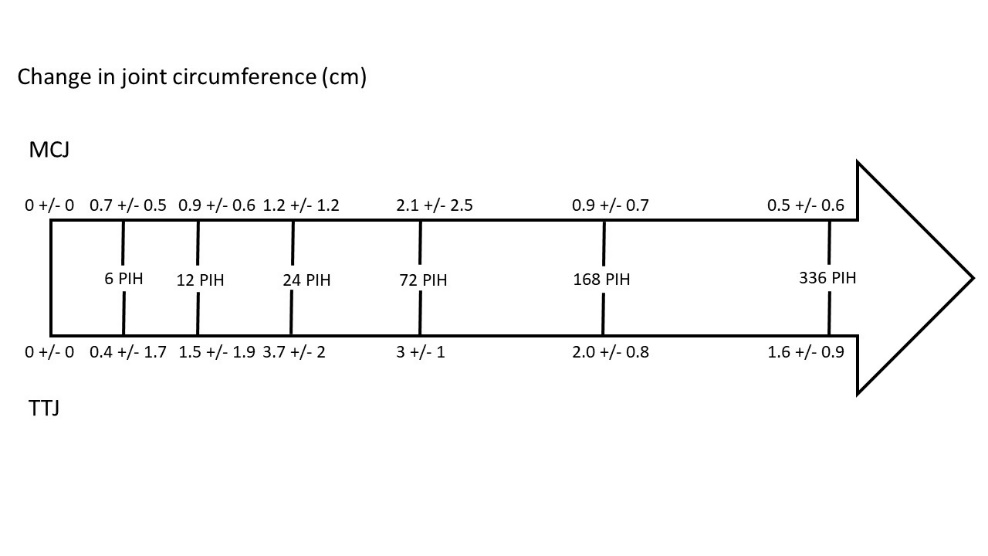


**Figure 6.** Progression of change in joint circumference (cm, mean +/- standard deviation) changes over 336 post-injection hours after administration of 75ng rIL-1β into the middle carpal joint (MCJ) and tibiotarsal joint (TTJ) of the horse.


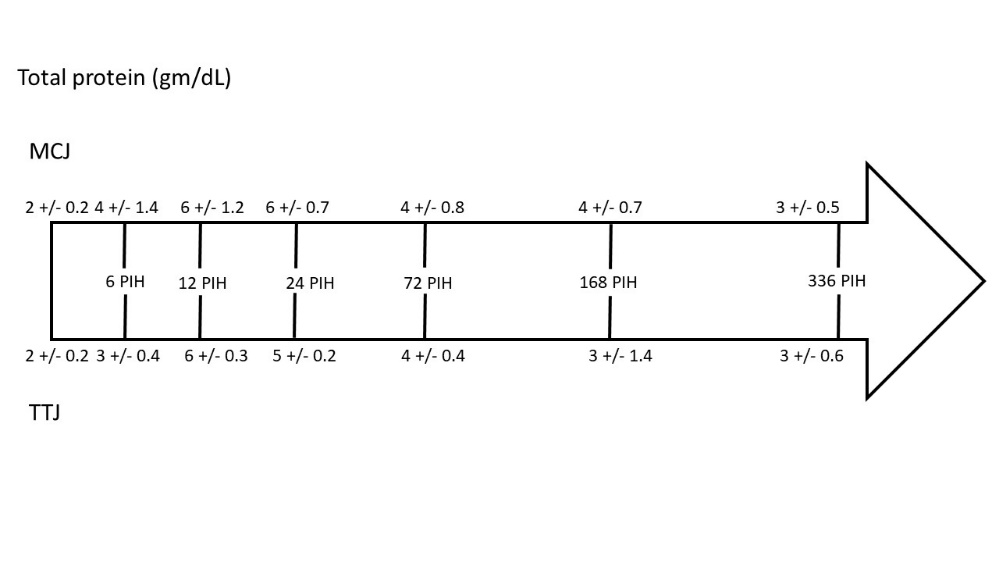


**Figure 7.** Progression of total protein of the synovial fluid (gm/dL, mean +/- standard deviation) changes over 336 post-injection hours after administration of 75ng rIL-1β into the middle carpal joint (MCJ) and tibiotarsal joint (TTJ) of the horse.


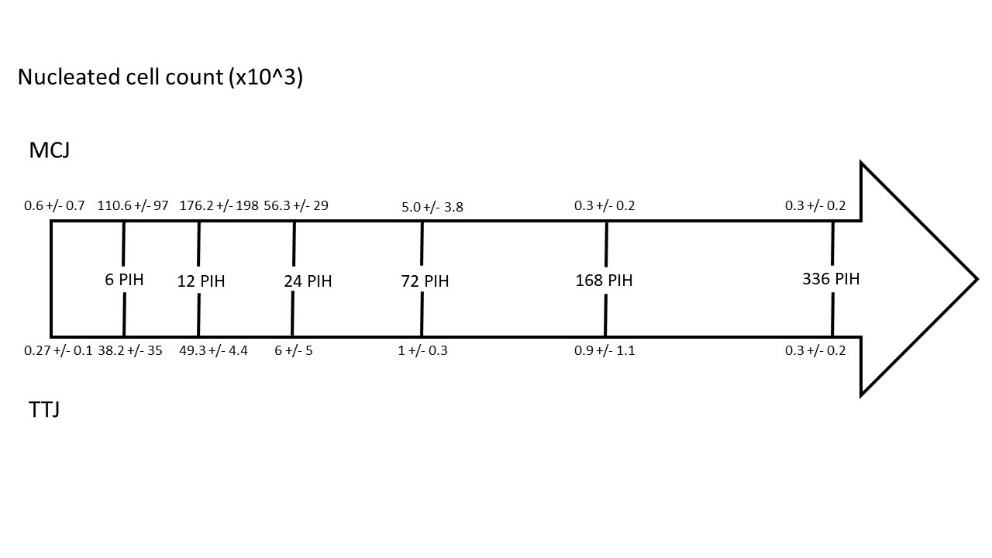


**Figure 8.** Progression of nucleated cell count in the synovial fluid (x10^3/uL, mean +/- standard deviation) changes over 336 post-injection hours after administration of 75ng rIL-1β into the middle carpal joint (MCJ) and tibiotarsal joint (TTJ) of the horse


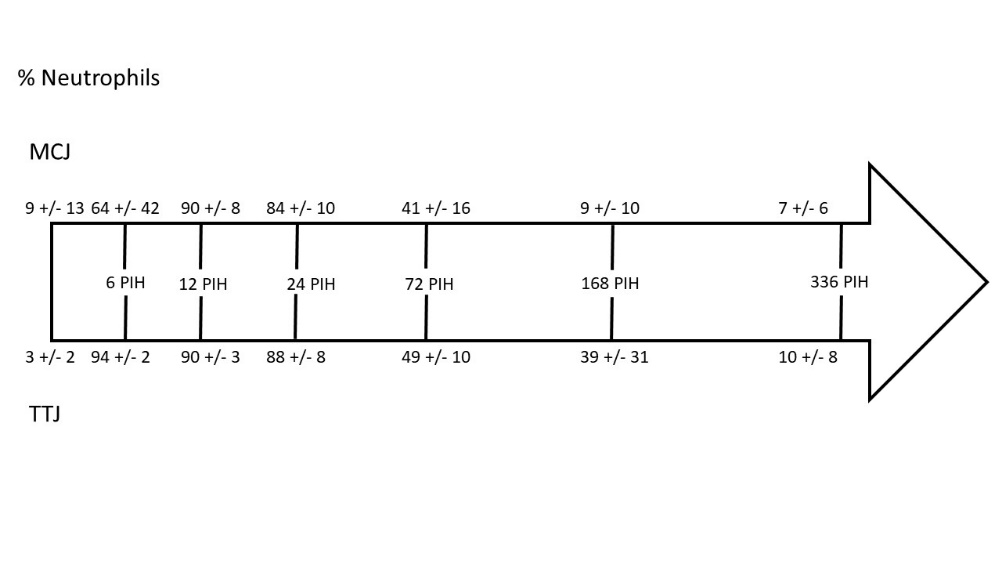


**Figure 9.** Progression of percent neutrophils within the synovial fluid (%, mean +/- standard deviation) changes over 336 post-injection hours after administration of 75ng rIL-1β into the middle carpal joint (MCJ) and tibiotarsal joint (TTJ) of the horse.
